# Underreported injection drug use and its potential contribution to reported increase in sexual transmission of HIV in Kazakhstan and Kyrgyzstan

**DOI:** 10.1186/s12954-018-0274-2

**Published:** 2019-01-05

**Authors:** Anna P. Deryabina, Padmaja Patnaik, Wafaa M. El-Sadr

**Affiliations:** 1 ICAP at Columbia University, 34/1 Samal -3, Almaty, 050051 Kazakhstan; 2 ICAP at Columbia University, Allan Rosefield Building, 722 West 168th Street, Room 1310, New York, NY USA

**Keywords:** People who inject drugs, Sex partners, PWID, Hepatitis C, HIV, Sexual transmission

## Abstract

**Background:**

We conducted a cross-sectional integrated bio-behavioral survey among sex partners of persons who inject drugs (PWID) to explore reasons for reported increase in reporting of heterosexually transmitted HIV in Kazakhstan and Kyrgyzstan.

**Methods:**

Sexual partners of PWID were recruited through PWID. Behavioral data were collected through semi-structured interviews. Dried blood spots were obtained and tested for HIV and hepatitis C virus antibodies (HCVAb). Descriptive univariate and bivariate analyses, and multivariate analyses using logistic regression modeling were performed to identify factors associated with HIV and HCV infections.

**Results:**

Among 1982 sex partners of PWID, overall HIV prevalence was 6.4%; 5.1% and 12.9% among those reported never and ever injecting drugs, respectively (*p* < 0.001). Overall, HCVAb prevalence was 21.3%; 15.0% and 53.9% among those reported never and ever injecting drugs, respectively (*p* < 0.001). Of HCV-positive participants, 58% and 34% (*p* < 0.001) reported prior history of injecting drug use among men and women, respectively. HIV prevalence was lower among HCV-negative (4.2%) compared to HCV-positive participants (14.4%) (*p* < 0.001). HIV prevalence was 3.5% (95%CI = 2.4–4.6) in a subset of female participants with no reported prior injecting drug use history and who were HCVAb-negative and did not report having an HIV-positive sex partner. Participant sex and number of sex partners as well as use of condoms in the past 12 months were not associated with HIV seropositivity.

**Conclusions:**

High prevalence of HCV among sex partners of PWID who denied ever injecting drugs suggests underreporting of injecting practices. The increased attribution of HIV infection to sexual transmission based on self-report may be partly explained by underreporting of injection drug use due to stigmatization of this behavior.

## Background

Central Asia remains one of the few regions in the world where the HIV epidemic is growing [1]. While HIV prevalence in the general population is less than 0.5%, the prevalence is above 5% in key populations, including persons who inject drugs (PWID) [2]. Injecting drug use has been noted as the main driver of HIV epidemics in the region; however, since 2010, national surveillance data from Kazakhstan and Kyrgyzstan suggest a steady increase in heterosexual transmission of HIV, based on self-report by newly detected HIV-infected individuals, especially among women [3]. In 2015, these national data collected as part of routine HIV case surveillance from HIV testing sites indicated that more than 50% of persons living with HIV (PLHIV) in both Kazakhstan and Kyrgyzstan were PWID [4] and that sex partners of PLHIV constituted a substantial proportion of newly detected HIV cases [3]. It is, thus, reasonable to assume that sex partners of PLHIV who are PWID are at increased risk for HIV infection and may constitute a growing percentage of newly detected HIV cases. At the same time, according to the national HIV testing data (also referred to as Form 4), testing yield among women tested for HIV as part of their antenatal care from 2010 to 2015 remained stable at 0.01% in Kazakhstan and at 0.03–0.04% in Kyrgyzstan.

The reasons for the reported increase in the number of reported cases of sexually acquired HIV infections registered in the national surveillance systems are not fully understood. The critical question is whether the increase in reported heterosexually acquired HIV among sexual partners of PWID who are HIV-infected is largely due to true increases in sexual transmission or due to underreported injection drug use by sexual partners who are PWID. No study to date has attempted to better understand the reasons behind the increased reporting of heterosexually acquired HIV infection in Kazakhstan and Kyrgyzstan.

To address this gap, we examined biological and behavioral characteristics of sex partners of PWID in order to assess possible reasons for reported increases in heterosexual HIV transmission in Kazakhstan and Kyrgyzstan.

## Methods

### Aim and design

We conducted a cross-sectional survey in a convenience sample of sex partners of PWID to determine HIV and hepatitis C virus (HCV) seroprevalence and to identify factors associated with HIV infection. Verbal informed consent was obtained from all participants.

### Setting

The data were collected during June–August 2013. The study was conducted in four cities in Kazakhstan (Karaganda, Temirtau, Ust-Kamenogorsk, and Kostanai) and four cities in Kyrgyzstan (Bishkek and Tokmok, Jalalabad and Osh) reporting the highest rates of sexually transmitted HIV infections as per the national case surveillance data in each of the two countries.

### Study population and sampling

We enrolled consenting females or males that were at least 18 years of age who reported a history of oral, anal, or vaginal sex with a PWID during the last 12 months. To limit to non-injecting persons, both people who reported injection drug use within the preceding 12 months and those who had visible marks of recent injections were excluded. Most participants were recruited directly through PWID, the latter via respondent-driven sampling to participate in routinely conducted national sentinel integrated biological behavioral surveys (IBBS). As part of the routine IBBS interviews, PWID were asked if they had non-injecting sex partners during the past 12 months. Following IBBS interviews, respondents that indicated having sex partners during the prior 12 months were asked to refer their non-injecting sex partners using recruitment coupons, depending on the number of sex partners reported during the interview. PWID participating in IBBS who brought their sex partners to participate in the survey were compensated with pre-paid mobile phone cards. Additional sex partners were recruited through PWID who visited non-governmental organizations (NGOs) working with PWID rather than during the IBBS. Sex partners of PWID who presented to the study sites were offered participation in the study.

A total of 1640 PWID (940 in Kazakhstan and 700 in Kyrgyzstan) included in the IBBS were approached for recruitment of their sex partners. On average, one to two recruitment coupons per PWID were provided (in total 2410 coupons were distributed). A total of 2055 sex partners of PWID presented to the study sites, and 2022 (98%) sex partners of PWID who met the selection criteria agreed to participate in the study. Of 2022, 1625 (80%) were recruited through PWID in the IBBS, while 397 (20%) were recruited through PWID visiting NGOs. Forty participants (2 in Kazakhstan and 38 in Kyrgyzstan), whose surveys were largely incomplete, were excluded from further analysis, thus reducing the final number of study participants to 1982 (1125 from Kazakhstan and 857 from Kyrgyzstan).

### Serology PWID

Presence of antibodies to HIV and HCV was ascertained by testing blood specimens collected from study participants through dried blood spot (DBS). Enzyme-linked immunoabsorbent assay (ELISA) was used in accordance with the national HIV (initial and confirmatory) and HCV testing algorithms approved for use in IBBS. The types of ELISAs used for initial testing varied by country for each of the two viruses, but confirmatory testing was done by Murex (ABBOTT) according to the manufacturers’ instructions. In Kazakhstan, samples testing positive by CombiBest anti-HIV 1,2 assay (Vektor-Best) were confirmed by Murex anti-HIV assay (ABBOTT). For HCV testing, samples that screened positive by Best anti-HCV assay (Vektor-Best) were confirmed by Murex anti-HCV ELISA (ABBOTT). In Kyrgyzstan, samples testing positive for HIV using the UniBest anti-HIV 1,2 assay (Vektor-Best) were confirmed by Murex anti-HIV assay (ABBOTT). For HCV testing, samples testing positive for HCV by RecombiBest anti-HCV-strip assay (Vektor-Best) were confirmed by Murex anti-HCV ELISA (ABBOTT).

### Sample size and statistical analysis

The target sample size was determined based on the average number of non-injecting sex partners per PWID per year as reported in the IBBS among PWID.

Following descriptive univariate and bivariate analyses, multivariate analyses were conducted using logistic regression modeling to identify factors independently associated with HIV and HCV, while adjusting for potential clustering in participants and their recruiter by using generalized estimating equations (GEE).

To estimate HIV prevalence among a subset of participants least likely to be injecting drugs, we conducted a sub-analysis among sex partners who reported no prior injecting use history and tested negative for HCVAb.

All analyses were conducted using Stata [5]. Pearson’s chi-square test was used to look at the magnitude of differences and judge the significance. Associations were assessed to be significant if *p* values were < 0.05. Full models (containing all the covariates) were tested for model fit using the Pearson’s goodness of fit test before model reduction for assessing independent associations.

## Results

### Social and demographic characteristics

Participants included 1706 women (86%) and 276 men (14%) ranging in age from 18 to 65 years with a median and mean age of 33 years (IQR = 27–39). Most male participants either had a cohabiting partner without being officially married (33%) or were single and not residing with a partner (31%), while female participants were either married (22%), had a cohabiting partner without being married (25%), were single and not residing with a partner (24%), or were divorced (22%). Responses to levels of income and contributions to family budgets also varied by sex with 43% of male participants responding that they were key income generators for their families, while only 24% of women gave this response (*p* < 0.001).

### Prior drug injecting behavior

By definition, all participants reported not having injected drugs in the 12 months preceding study entry. Among the latter group, most participants (83.6%; 95%CI 81.9–85.2) reported never injecting drugs. Reports of ever injecting drugs were significantly higher among male partners (38.8%; 95%CI 33.0–44.6) compared to female partners (12.8%; 95%CI 11.2–14.4) (*p* < 0.001).

### Sexual behavior and history of sexually transmitted infections

Regarding the number of sex partners over the past 12 months, the majority of men (201 participants, 72.8%) reported more than one partner, while 42.1% women (718 participants) did so (*p* < 0.001). The majority (836 participants, 91.0%) of participants with more than one sex partner in the last 12 months reported having had sex with both PWID and with individuals who did not inject drugs. Of all participants, in the past 12 months, 30.0% (95%CI 28.0–32.0) reported that they always used condoms with their partners who inject drugs and 28.1% (95%CI 25.0–31.0) of those who had both PWID and non-injecting sex partners reported always using condoms with non-injecting partners (*p* > 0.05). Use of condoms with different types of partners did not differ by sex (*p* > 0.05) (Table 1).

**Table 1:** Selected sexual behaviors reported in the preceding 12 months

	Men (*N* = 276) % (95% confidence interval)	Women (*N* = 1706) % (95% confidence interval)	*p*-value
Reported more than one sex partner	72.8% (67.5–78.1)	42.1% (39.7–44.4)	< 0.001
Use of condoms with PWID partners			
Always	32.6% (27.1–34.5)	29.5% (27.4–31.8)	0.30
Sometimes	38.8% (33.0–44.8)	43.1% (40.8–45.5)	0.30
Never	28.6% (23.4–34.3)	27.3% (25.2–29.5)	0.65
Use of condoms with non-PWID partners among respondents who reported having sex with both PWID and non-PWID partners in the preceding 12 months (*N* = 196 men and 650 women)			
Always	30.6% (24.2–37.6)	27.2% (23.8–30.8)	0.36
Sometimes	41.3% (34.4–48.6)	52.3% (48.4–56.2)	0.36
Never	28.1% (21.9–34.9)	20.5% (17.4–23.8)	0.35

Reports of symptoms of sexually transmitted infections (STIs) in the past 6 months were significantly higher among female participants (23.4%; 95%CI 21.4–25.4) compared to male participants (9.8%; 95%CI 6.2–13.3) (*p* < 0.001). Frequency of condom use was not associated with report of STI symptoms. However, women who reported more than one sex partner over the past 12 months and had history of ever injecting drug use in the past, i.e., prior to the last 12 months, were more likely to report STI symptoms in the past 6 months.

### HCV prevalence

HCV seroprevalence among participants was high (21.3%) and significantly higher among men compared to women (48.9% in men and 16.9% in women, (*p* < 0.001). Overall, HCV seroprevalence was significantly higher among those who reported more than one sex partner in the last 12 months (23.5% vs 19.5%, *X*^2^ (1) = 4.77, *p* = 0.03), and this was noted similarly among women and men. HCV prevalence was significantly higher among participants who had ever injected drugs in the past (72.9% in men and 44.5% in women) compared to those who reported never injecting drug use (33.7% in men and 12.8% in women), *p* < 0.001). The history of ever injecting drugs was the only significant predictor of HCVAb positivity in men and women (OR = 6.6; 95%CI = 5.1–8.5) (Table 2); however, only 57.8% of HCVAb-positive men and 33.6% of HCVAb-positive women (*p* < 0.001) reported prior history of ever injecting drugs. Of 245 HCVAb-positive women with HIV, 68.2% reported never injecting drug use (95%CI 61.9–74.0).

**Table 2:** Factors and their association with HCV seropositivity among participants based on multivariate analyses (*N* = 1982)

	Adjusted odds ratio (aOR)	*p*-value	95% confidence interval	
History of ever injecting drug use in the past	5.41	< 0.001	4.14	7.05
Male gender	3.48	< 0.001	2.58	4.70
More than one sex partner in the past 12 months	0.95	0.68	0.75	1.21
Reported always using condoms during the past 12 months	0.90	0.49	0.68	1.20

In the subset of 1572 participants (79% of all study participants) with no reported prior injecting drug use history that were also HIV-negative, HCV prevalence was 11.8% in women and 31.2% in men (*p* < 0.001).

### HIV prevalence

Overall, HIV prevalence among participants was 6.4% and marginally higher among men (9.1%) compared to women (6.0%) (*p* = 0.05). Irrespective of sex, HIV prevalence was significantly lower among those who reported no history of injecting drug use (5.1%; 95%CI 4.1–6.3) versus those who had ever injected drugs in the past (12.9%; 95%CI 9.5–17.1) (*p* < 0.001). HIV prevalence was significantly lower (4.2%) in participants who were HCV-negative compared to HCV-positive participants (14.4%) (*p* < 0.001). There was no statistically significant difference in HIV prevalence by number of sex partners in the past 12 months.

A limited number of participants (31 females and 6 males; 2% of study population) reported being aware of having an HIV-positive partner. For female participants, having an HIV-positive partner and their own prior use of injecting drugs were two factors associated with significantly higher HIV-positive prevalence (*p* < 0.001), but the number of sex partners (*p* = 0.37), reported STI symptoms (*p* = 0.05), and the reported consistent use of condoms with all partners over the past 12 months (*p* = 0.53) were not associated with seropositivity. For male participants, having an HIV-positive partner and the reported consistent use of condoms with all partners over the past 12 months were the only factors associated with higher risk of HIV seropositivity (*p* < 0.001).

In the subset of 1103 female participants (56% of all study participants; 65% of all female participants) with no reported prior injecting drug use history that were also HCV-negative and did not report having an HIV-positive sex partner, HIV prevalence was 3.5% (95%CI = 2.4–4.6). In the final logistic regression model, the number of sex partners, reporting consistent use of condoms and having STI symptoms, was not significantly associated with HIV seropositivity.

## Discussion

This study demonstrated that both HIV and HCV prevalence among female sex partners of PWID was significantly higher than that estimated in the general population of women and other populations, like blood donors (less than 0.5% for HIV and less than 5% for HCV), in Kazakhstan and Kyrgyzstan [6–9]. Among female sex partners, reported history of prior injection drug use and of having an HIV-positive sex partner who injects drugs was associated with HIV infection. Among male participants, reported consistent use of condoms was associated with HIV seropositivity; however, this may be due to the fact that men who are aware of their HIV-positive status or the HIV-positive status of their partner were more likely to use condoms as shown by several other studies [10–12].

The study identified, unsurprisingly, that having an HIV-positive male sex partner who injects drugs was associated with HIV infection among female participants who had no history of drug injection. In addition, as PWID in Kazakhstan and Kyrgyzstan account for the largest proportion (more than 50%) of all confirmed HIV cases, having sex with a PWID presents a substantial risk for sexual transmission of HIV. Alarmingly, less than one third of women participants in our study reported consistent use of condoms with their PWID partners over the past 12 months. This finding is similar to the results reported from other studies [13–15]. It is likely that the sexual transmission from HIV-positive PWID to their sex partners is facilitated by the fact that the majority of PWID who are HIV-positive are likely unaware of their HIV-positive status and, if aware, are unlikely to be enrolled in care or have initiated antiretroviral therapy or achieved viral suppression [16], consequently enhancing the risk of HIV transmission [17].

An intriguing finding from our study is the high HIV prevalence (4.4%) among sex partners of PWID who reported never injecting drugs and who did not report having an HIV-positive PWID sex partner. This finding is consistent with results from another study conducted among sex partners of PWID in Almaty, Kazakhstan, which showed 10.4% HIV prevalence among female partners who reported never injecting drugs and the authors in the latter study assumed that HIV infection in this group occurred heterosexually [18]. In our study, HCV seroprevalence in the subset of sex partners of PWID who reported never injecting drugs and who did not report having an HIV-positive PWID sex partner was also high (13%). HCV is very effectively transmitted by injections and has been used as a biomarker for injecting-related risks [19, 20]. Sexual transmission of HCV does occur, although rarely, among HIV-infected women and HIV-infected MSM [21, 22]. In other populations, sexual transmission of HCV in heterosexual couples is unlikely [23–25], and therefore, HCV infection can serve as a marker of parenteral transmission. This marker may be quite useful as earlier studies showed that self-reported information about injection drug use can be unreliable, usually underestimating such behavior, especially among women who often avoid disclosing their drug injection history due to fear of stigma [26]. Thus, it is interesting to note that only a third of all HCV-positive women and almost 70% of all HCV-positive women without HIV infection in our study reported no prior history of injecting drug use, suggesting possible underreporting of injecting behavior. The absence of significant changes in the rates of HIV prevalence among pregnant women in both countries rules out increased antenatal screening as a cause of increased reporting of heterosexually acquired HIV, thus supporting the possibility that the reported increased prevalence of heterosexual transmission may be due to underreporting of injecting behavior.

The study had several limitations. It was conducted in select cities in Kazakhstan and Kyrgyzstan, which have the highest reported rates of HIV transmitted through sex; therefore, the survey results cannot be fully extrapolated to all regions or to Kazakhstan and/or Kyrgyzstan. In addition, the sampling of sex partners was not random, which may have influenced the prevalence of HIV and HCVAb. Additionally, ascertainment of drug use was dependent on self-report and examination for evidence of active drug use such as signs of intoxication or fresh injection marks does not exclude history of injection drug use. Lastly in our study, for prior sex behaviors, participants were asked to report on the number of sex partners during the past 12 months, which differs from the usual recall period used in studies of sex behavior. A meta-analysis that included 28 studies [27] showed that a recall period of 6 months was best for recalling a number of sex partners.

## Conclusion

HIV and HCV prevalence were alarmingly high among reportedly non-injecting sex partners of PWID in Kazakhstan and Kyrgyzstan. The high prevalence of HCV suggests possible underreporting of previous or current injection drug use, especially among women, likely due to fear of stigma and the need to provide socially desirable responses to questions regarding route of HIV acquisition. The study is unable to determine the magnitude of the effect of underreporting of injecting behaviors on the number of HIV-positive individuals who report sexual transmission as their risk factor for HIV acquisition. More accurate data on risk behavior for HIV acquisition will require training of surveillance officers in how to gain the confidence of the respondents, assuring them confidentiality of all reported information and the inclusion of HCVAb testing among individuals identified with HIV infection.

Programs for the prevention of HIV are urgently needed for PWID and their sex partners. Such programs should address both safer injecting drug use and safer sex behaviors in both groups. Establishing and scale-up of accessible programs for HIV testing and counseling, including rapid HIV testing, linkage of those found to be HIV-positive to care with prompt initiation of antiretroviral therapy are urgently needed, especially among those in discordant partnerships, with focus on achieving and maintaining viral suppression. In addition, scale-up of harm reduction programs including opioid agonist therapy is critical to reduce HIV transmission and improve engagement and adherence with HIV prevention and treatment which is of paramount importance to control the HIV epidemic in this region of the world.

## Abbreviations

CDC: Centers for Disease Control and Prevention
DBS: Dried blood spot
ELISA: Enzyme-linked immunoabsorbent assay
HCV: Hepatitis C virus
HIV: Human immunodeficiency virus
IBBS: Integrated biological behavioral survey
PLHIV: Persons living with HIV
PWID: Persons who inject drugs
